# Robotics and AI into healthcare from the perspective of European regulation: who is responsible for medical malpractice?

**DOI:** 10.3389/fmed.2024.1428504

**Published:** 2024-09-06

**Authors:** Francesco De Micco, Simone Grassi, Luca Tomassini, Gianmarco Di Palma, Giulia Ricchezze, Roberto Scendoni

**Affiliations:** ^1^Research Unit of Bioethics and Humanities, Department of Medicine and Surgery, Università Campus Bio-Medico di Roma, Rome, Italy; ^2^Operative Research Unit of Clinical Affairs, Fondazione Policlinico Universitario Campus Bio-Medico, Rome, Italy; ^3^Forensic Medical Sciences, Department of Health Sciences, University of Florence, Florence, Italy; ^4^School of Law, Legal Medicine, Camerino University, Camerino, Italy; ^5^Department of Public Health, Experimental, and Forensic Medicine, University of Pavia, Pavia, Italy; ^6^Department of Law, Institute of Legal Medicine, University of Macerata, Macerata, Italy

**Keywords:** robotics, artificial intelligence, medical liability, medical negligence, clinical decision making, accountability, tort law

## Abstract

The integration of robotics and artificial intelligence into medical practice is radically revolutionising patient care. This fusion of advanced technologies with healthcare offers a number of significant benefits, including more precise diagnoses, personalised treatments and improved health data management. However, it is critical to address very carefully the medico-legal challenges associated with this progress. The responsibilities between the different players concerned in medical liability cases are not yet clearly defined, especially when artificial intelligence is involved in the decision-making process. Complexity increases when technology intervenes between a person’s action and the result, making it difficult for the patient to prove harm or negligence. In addition, there is the risk of an unfair distribution of blame between physicians and healthcare institutions. The analysis of European legislation highlights the critical issues related to the attribution of legal personality to autonomous robots and the recognition of strict liability for medical doctors and healthcare institutions. Although European legislation has helped to standardise the rules on this issue, some questions remain unresolved. We argue that specific laws are needed to address the issue of medical liability in cases where robotics and artificial intelligence are used in healthcare.

## 1 Introduction

Artificial intelligence (AI) and robotics have joined forces, heralding an exhilarating and unstoppable era. Among the sectors witnessing captivating applications of robotics, healthcare stands out, solidifying its status as a domain where the integration of cutting-edge technologies has yielded significant breakthroughs (1, 2).

AI is a set of computational techniques inspired humans use their own nervous system and body to sense, learn, reason, and act (3). Robotics is the AI in action in the physical world, also known as embodied AI. Robots are physical machines designed to address the dynamics, uncertainties, and complexities of the physical world. In robotic systems, the control architecture typically integrates capabilities for perception, reasoning, action, learning, and interaction with other systems (4).

By 2021, 42% of healthcare organisations in the European Union had already integrated artificial intelligence technologies for disease diagnosis, demonstrating a growing trend in the adoption of innovative solutions in the medical sector. In addition, a further 19% of these organisations planned to implement such technologies within the next 3 years, showing a strong inclination towards the use of AI to improve diagnostic accuracy and efficiency. At the same time, 39% of healthcare organisations planned to adopt AI-based patient monitoring tools within the same period, aiming to improve the ongoing management and monitoring of patient health, thereby optimising clinical outcomes and enhancing early intervention capability. Furthermore, 28% of organisations were already using robotics and a further 25% were planning to implement robotics-based healthcare solutions. These figures underline the growing importance of AI in healthcare and its potential transformative impact in the coming years (5, 6).

The applications of robotics in the healthcare scenario are manifold and encompass areas such as, diagnosis, therapy, and rehabilitation (7).

In the field of medical care, robotic systems allow remote patient examination, advanced diagnosis and monitoring of vital parameters (8, 9). This includes smart medical capsules, technological devices designed to administer drugs and monitor various biological parameters within the human body. The capsules are equipped with sensors, actuators and communication technologies that enable them to interact with the body and transmit real-time data to doctors or external monitoring systems (10–13).

In the therapeutic area, robotics is most widely used in robot-assisted surgery, in which human activity is supported by technological instruments capable of performing remote-controlled operations. Robot-assisted microsurgery provides a higher level of precision, eliminates the physiological tremor of the surgeon’s hand and possible iatrogenic injuries, but compared to the surgeon’s hand has less adaptability to soft tissue (14–16); precision robotic surgery being able to define, plan and process 3D models of the patient allows the autonomous execution of pre-programmed surgical tasks on “hard” materials such as bone (17, 18); minimally invasive robotic surgery increases the precision of surgical procedures and the speed with which they are performed, shows the surgical field in high definition, eliminates the physiological tremor of the surgeon’s hands, and thanks to the ergonomics of the console, provides greater comfort for the surgeon. However, in addition to high costs, it requires high skills and special training (19–22); an interesting area for the prospects it may have is that of telesurgery, which allows remote surgical interventions such as the well-known “Lindberg Operation” in which a robot-assisted laparoscopic cholecystectomy was performed by a remote surgeon more than 14,000 km away from the patient’s operating table (23). The 5G integration of telesurgery can expand the skills of the remote surgeon due to the high-speed, low-latency connectivity offered by the network and the possibility of using augmented reality (24).

There are the applications of robotics in the area of rehabilitation, to which robot-assisted rehabilitation, robot-assisted mental, cognitive, and social therapy and robot-assisted mobility systems belong. After injuries to the central nervous system that impair motor coordination, recovery of motor function and skills involves repeated movement of the affected part and stimulation of brain plasticity. Robotics applied to rehabilitation facilitates guided movement of the upper and lower limbs, optimising therapeutic and functional effects. These technologies also provide feedback to the patient, allowing the force to be adjusted and thus maximising the effectiveness of the therapy, stimulating the recovery process (25–29). Robot-assisted rehabilitation offers muscle support therapies and repetition of basic motor activities, allowing users to perform them comfortably in the home environment through integration with personal computers. These tools often make use of technologies originally developed for other purposes, such as gaming (30–32). Robot-assisted mental, cognitive and social therapy systems are designed to interact with humans, simulating different types of social behaviour, such as communication and cooperative play. These robotic tools are applied in patients suffering from dementia, Alzheimer’s disease, autism, and children’s motor disabilities (33–36). Robotic wheelchairs, smart walkers, and exoskeleton are robotic mobility aid systems as alternatives to traditional tools for patients with severe motor difficulties (37, 38).

Finally, the use of Large Language Models (LLM) in the healthcare field must also be considered. Language models like ChatGPT show potential as virtual assistants in radiology, helping to streamline various tasks, but they present significant limitations. The latest version (GPT-4) cannot interpret medical images and its recommendations can be inaccurate, requiring the professional judgement of radiologists. Integration with electronic health record (EHR) systems poses challenges of privacy and coordination (39).

The integration of robotics in healthcare can lead to several benefits, including faster execution of procedures, improved diagnostic accuracy and increased efficiency in clinical operations. In addition, robotics offers the possibility of performing examinations and operations even for individuals who would otherwise be unable to access them due to geographical, political or economic limitations.

Delineate the applications of robotics and AI in healthcare and their potential, it is also necessary to highlight that there are legal and ethical challenges related to privacy and data protection, informed consent, the creation of possible new inequalities in access, the ethical implications of algorithmic decisions, and the identification of responsibilities in the event of an error committed by a surgical robot or an AI system (40, 41). Although it is often not possible to separate ethical and legal issues into distinct compartments due to their multidirectional interconnections, the topic of responsibility is the focus that this manuscript intends to address.

**Figure 1:**
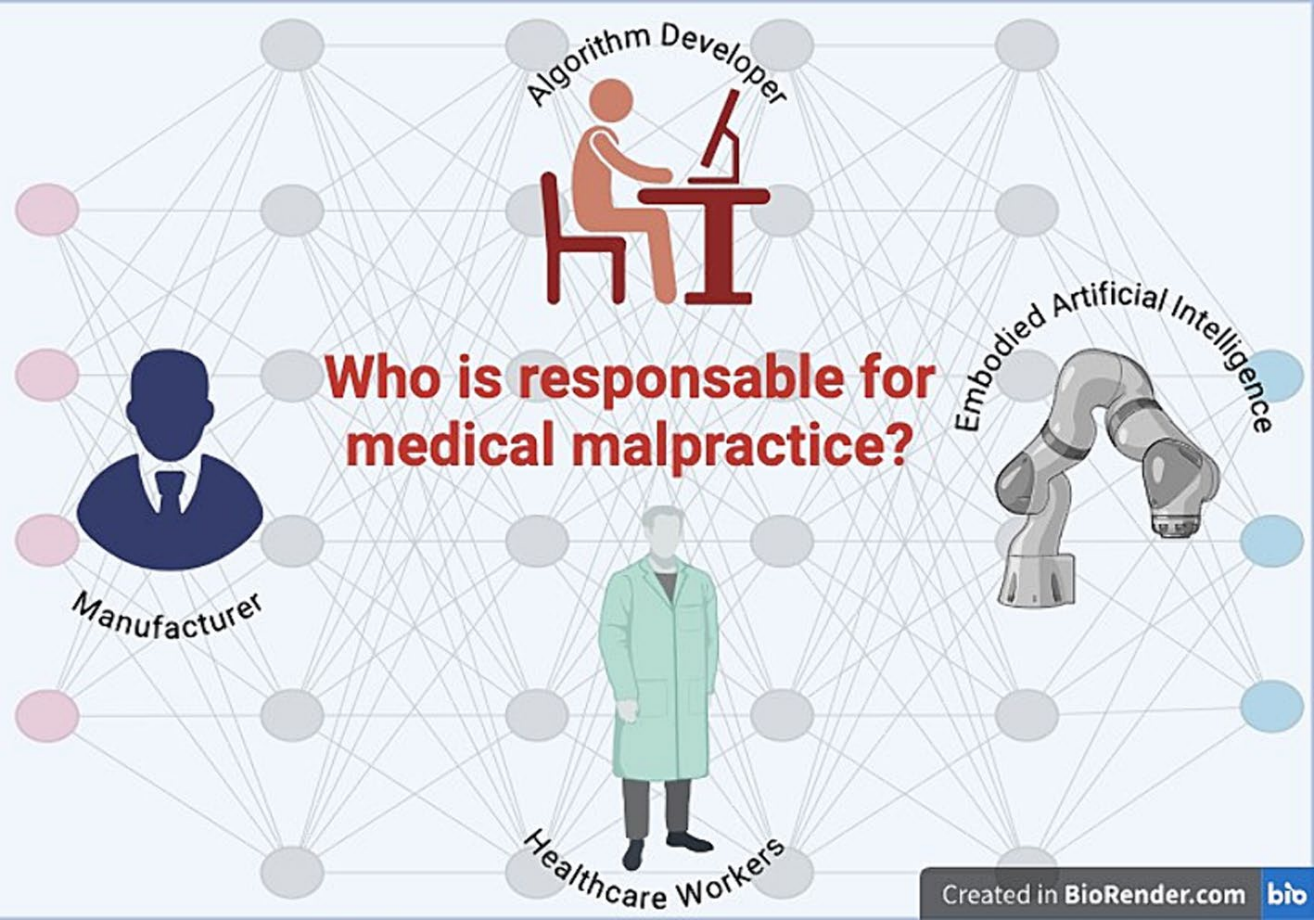
Human and non-human beings to whom medical negligence could be attributed in a robotic and AI-based care context.

A robotized care process could reduce the responsibilities borne by the subjects normally involved in a non-robotized care process. If this were true, how and in what terms could the error committed by the robot be complained about? Who is responsible in case of errors or damages caused by a medical robot? If the robot is a consumer product, product liability law may apply. This question becomes even more intricate when considering that robots can operate autonomously or semi-autonomously (42–44). Liability could also extend to the developer of the software or algorithm. The answer is not so simple and immediate. Who will be liable for the tort committed by the autonomous robot-agent? Traditionally, legal liability has been attributed to human beings, who can be held responsible for their actions under the law. In this context, can the manufacturer of the robot be held liable for the tort committed by the autonomous robot? Or, could the manufacturer be exempt from liability if it can prove that it was unable to entirely foresee the robot’s actions. This raises the question of how to establish the degree of reasonable foresight that can be expected from the manufacturer of an autonomous robot. Is the liability of Healthcare Workers (HWs) limited to errors caused by misuse or mismanagement of the robot? (Figure 1).

With the increasing integration of AI and robotics into clinical practice, it is essential to examine how regulations are adapting to address emerging challenges and ensure adequate protection for patients and healthcare professionals. In this rapidly evolving context, regulations and official reports play a crucial role in ensuring that the adoption of AI and new technologies occurs in a safe, ethical, and legally compliant manner. The European overview of regulations and official reports for AI and new technologies in healthcare will provide a valuable framework of the norms and guidelines shaping the use of these advanced technologies in a healthcare setting.

We will focus on the concept of “electronic persons,” which refers to the legal consideration of artificial entities such as robots and AI systems within the legal context. The status of electronic persons raises fundamental questions regarding their legal responsibility, rights, and duties, and represents an advanced frontier in technology law. Examining this status is crucial for understanding how laws and regulations must evolve to include new forms of artificial intelligence and robotics.

Therefore, we will address the issue of strict liability, which is central to the legal and ethical debate surrounding the use of advanced technologies in healthcare. Strict liability implies that a party can be held responsible for damages caused by technologies, such as surgical robots or AI systems, regardless of proof of fault or negligence. This form of liability is particularly relevant in the healthcare context, where technological errors can have serious and complex consequences. Exploring how strict liability norms apply to new technologies is essential for ensuring a fair and effective legal system.

In this article, we will examine in detail the European regulations and official reports regarding AI and new technologies in healthcare, analyze the concept and implications of the status of electronic persons, and discuss the implications of strict liability in the context of healthcare technology. The goal is to provide a comprehensive understanding of how regulations and legal issues intersect with technological innovation, thereby addressing the question: who is responsible for medical malpractice?

## 2 Regulations and official reports for AI and new technologies in healthcare: an European overview

Considering the complexity of the technology, the Scientific Foresight Unit (STOA) of the European Parliament Research Service (EPRS) argued that the EU legal framework needed to be updated, developing legislation based on risk analysis and making specific changes on a case-by-case basis. It was proposed that a code of conduct be established to set ethical standards to which researchers, practitioners, users and designers should adhere (45).

On 17 February 2017, the European Parliament called on the Commission to submit a legislative proposal to establish civil law rules on robotics and AI (46). Unlike other legislative resolutions, this parliamentary initiative merely laid down a set of principles. The resolution defined intelligent robots as machines capable of acquiring autonomy by interacting with their surroundings through sensors and exchanging data. This process allows them to analyse crucial information. Furthermore, these robots can learn from past events and interactions, and their physical form provides the necessary tangible support. Finally, the ability to adapt behaviour and actions to their surroundings completes the picture of their powerful capabilities. The introduction of robots in healthcare should not impair the doctor-patient relationship, but provide the physician with assistance in diagnosis and/or treatment in order to reduce the risk of human error and increase quality and life expectancy. Nevertheless, the threat of the dehumanisation of care and the need to preserve the role of HWs due to the irreplaceability of the human factor in social interaction is felt. The importance of adequate education, training, and preparation is therefore emphasised, with the need to define the minimum professional requirements to be able to use surgical robots.

However, the resolution is particularly original and significant when it proposed the recognition of legal personality for robots that make autonomous decisions or interact independently with third parties so that these “electronic persons” can compensate for any damage caused by them. From this perspective, a joint human-robot action is recognisable based on the predictability and directionality of two interdependent relationships, the human and the robotic, and responsibility should be proportional to the actual level of instructions given to the robot and the latter’s degree of autonomy. Opposite to these statements is the position expressed by the European Economic and Social Committee (EESC), which called the introduction of a form of legal personality for robots or AI an unacceptable moral hazard. Among other things, the transfer of liability from the manufacturer to the robot could lead to the loss of the preventive function of correcting behaviour and to inappropriate use or abuse of the legal status (47).

In 2018, the European Union (EU) recognised the need to set high standards for AI-equipped systems in terms of safety and product liability, ensuring an appropriate legal framework (48). As regards the protection of personal data, the Commission specified that data subjects have the right to receive meaningful information on the logic used in the decision. In order to ensure fair and transparent data processing, the data subject must be provided with information on the logic used in automated decision-making as well as the possible consequences (49). Therefore, AI systems should be developed to enable humans to understand their actions and the underlying logic in order to increase transparency and minimise the risk of bias or error (50). In the document attached to the communication, the European Commission addressed the issue of liability for emerging digital technologies and stated that AI-based robots must meet the essential health and safety requirements set out in the EU regulations on machinery, radio equipment and medical devices as well as the directive protecting the health and safety of workers. Nevertheless, the limitation of the aforementioned regulations is recognised when a liability judgement must be made in situations where the damage is caused by an autonomous and self-learning technology. An example is the case of fully autonomous cars, for which it has been argued that liability for damage can be attributed to the driver/owner of the vehicle under tort law or to the manufacturer of the automated vehicle under the rules implementing the Product Liability Directive. Liability is based on fault or risk, where the holder/driver is strictly liable for opening the risk associated with the circulation of a motor vehicle on public roads (48).

Due to the lack of a specific regulatory framework concerning liability and insurance in the field of robotics and AI, the European Parliament proposed to introduce a harmonised European regulatory framework to enable a tailor-made approach to robotics and AI. On the contrary, it is considered unsatisfactory not to develop additional measures to the existing regulatory framework or to extend the scope of the Product Liability Directive (51).

Subsequently, the Commission proposed to pursue an AI in the service of people with the ultimate goal of improving the well-being of human beings. An “anthropocentric” AI should provide for the subsistence of surveillance mechanisms, safety devices and traceability. Surveillance could be ensured by adopting an approach with human intervention (human-in-the-loop), with human supervision (human-on-the-loop) or with human control (human-in-command); safety devices should be incorporated from the design phase, to ensure the safety of AI systems in a verifiable manner during each phase, with particular attention to the physical and mental protection of all individuals involved; the algorithm used should be described and the decision-making process should be explained, recorded and documented. Finally, in the event of an unfair negative impact, accessible mechanisms should be provided to ensure adequate means of redress (52).

In 2020, the European Commission issued a white paper on AI that investigated the challenges and opportunities associated with AI and proposed guidelines for its ethical and sustainable development. According to this official report, although software intended to be used for medical purposes must be considered a medical device under the Medical Devices Regulation, two critical issues remain to be considered: whether stand-alone software can fall within the scope of EU product safety legislation and whether EU product safety legislation can also be valid and sufficient for AI-based services, such as healthcare services. The need to adapt the legal framework to digital transformations and the use of AI requires specific regulatory interventions, the drafting of which will have to be a priority following a risk assessment based on two cumulative criteria. Before anything else, we must carefully assess the domain in which AI is applied, particularly highlighting healthcare as a field where significant risks are foreseeable due to the nature of typical activities. Secondly, the way in which AI is used in the sector under consideration: a possible defect in the appointment booking system in a hospital does not present a significant risk, but AI systems that provide medical information directly to the patient or AI systems that perform medical functions on a patient may be burdened with the risk of patient injury or death.

In the first case, the risks do not justify legislative intervention, whereas in the second case, the potential impact on individual rights warrants an adjustment of the legislative framework by introducing provisions that explicitly address the new risks arising from emerging digital technologies aimed at ensuring legal certainty (53).

The European Parliament Resolution of 20 October 2020 also regulated liability for AI system users on the basis of a risk assessment approach. An AI system that works autonomously can endanger the user or the public at random and to a much greater extent than can reasonably be expected is at high risk. A strict liability is identified for high-risk AI systems, whereby users are liable for any damage or harm resulting from the use of the system, excluding only cases of force majeure. Operators cannot excuse themselves by claiming that they acted diligently or that the damage was caused independently by the AI system. It applies both to “front-end operators,” i.e., the natural or legal person who exercises a degree of control over, and benefits from, a risk associated with the operation and functioning of the AI system, and to “back-end operators,” i.e., the natural or legal person who, on an ongoing basis, defines the characteristics of the technology and provides the essential back-end data and support service and therefore also exercises a high degree of control over a risk associated with the operation and functioning of the AI system. Both operators will have to verify the existence of civil liability insurance coverage appropriate to the amounts and scope of compensation required by the resolution: up to a maximum amount of EUR 2 million in the event of death or damage to health or physical integrity; up to a maximum amount of EUR 1 million in the event of property damage. The Resolution sets the limitation periods at 30 years from the date on which the damage occurred for personal injuries, 10 years from the date on which the damage occurred or 30 years from the date on which the harmful activity of the AI system took place for property damage. For AI systems that are not high-risk, a fault-based liability regime is identified. Whereas it is not possible to exonerate oneself from liability by claiming that the damage was caused independently by the AI system, the operator may instead prove that he is not liable if he proves that the damage was not related to a negligent action. This can occur if the AI system is activated without the operator’s knowledge, provided that all reasonable security measures have been taken. Furthermore, the operator may be considered not liable if they demonstrate having diligently selected an appropriate AI system, implemented it correctly, monitored its activities closely, and maintained operational reliability by regularly installing all available updates. With regard to the apportionment of liability, the operator sees its degree of liability decrease when the damage is the result of a contribution from both the AI system and the conduct of the injured party or another party for which the injured party is responsible. If there are several operators in the AI system, they are jointly and severally liable. If the “back-end operator” is also the producer, the Product Liability Directive applies; if the “front-end operator” is also the manufacturer of the AI system, the Product Liability Directive Resolution prevails (54).

On 13 March 2024, the European Parliament approved the AI Act aimed at ensuring the smooth functioning of the EU market by harmonising the rules for placing on the market, commissioning and use of AI systems. Using a risk-based approach, AI systems are differentiated into unacceptable risk, high risk and low or minimal risk. The use of AI systems that violate the fundamental values and rights of the EU is deemed unacceptable. This encompasses those posing a risk of manipulating individuals through subliminal techniques, exploiting specific vulnerable groups, contravening current legislation on data protection, consumer protection, and digital services. Additionally, assigning a social score based on AI for general purposes by public authorities and conducting real-time remote biometric identification in public spaces for law enforcement purposes are considered unacceptable, with certain limited exceptions. At high risk are AI systems intended for use as safety components of products subject to prior conformity assessment by third parties, as well as stand-alone AI systems that primarily have fundamental rights implications. In healthcare, this category includes AI systems used by or on behalf of public authorities to assess the eligibility of individuals for public assistance benefits and services, and to grant, reduce, withdraw or recover such benefits and services; Also included in this category are AI systems used to dispatch emergency first aid services or to prioritise the dispatch of such services. However, the list may be supplemented by adding AI systems that, in addition to falling within one of the areas already considered, also present an equivalent or higher risk of harm to health and safety than the risk of harm presented by AI systems already considered high risk. A risk management system is established for high-risk AI systems through which risks are identified, analysed and estimated, and measures are put in place for the mitigation and control of non-eliminable risks or the eradication of eliminable ones. In Article 14, the regulation deals with the human-machine interface, outlining the primary role of the human being in the decision-making process. Human surveillance will have to be planned by the provider prior to marketing and integrated into the high-risk AI system. The human being will be responsible for monitoring the functioning of the high-risk AI system, intervening promptly in the event of anomalies, malfunctions and unexpected performance, shutting down the system in good time. Furthermore, the human being must be aware of the risk of “automation bias,” i.e., an excessive and uncritical reliance on the output and the need to interpret and possibly disregard the output of the high-risk AI system. For AI systems already classified as high-risk, the execution of the action must be verified and confirmed by at least two human beings. Finally, high-risk AI systems are designed and developed to ensure that their operation is transparent to the extent that users can interpret the output of the system and use it correctly (55).

The aim of the AI Act is to prevent, monitor and address the risks associated with the use of AI, but it does not include measures for the benefit of people who have been harmed by it. Therefore, the European Parliament and the Council have proposed an AI Liability Directive that aims to ensure that persons claiming compensation for damage caused by an AI system have a level of protection comparable to that guaranteed to individuals claiming compensation for damage caused without the intervention of an AI system (56). To this end, the Proposal addresses issues concerning disclosure of evidence and the burden of proof with specific reference to claims for compensation concerning damage caused by the output produced by an AI system or the failure of that system to produce an output through the fault of a person. With regard to the disclosure of evidence, in order for the alleged injured party to assess the validity of a claim for compensation, interested parties should be granted the right to request a court to order the disclosure of relevant evidence before making a claim for compensation for the damage suffered. Accordingly, in the event that the defendant in an action for damages fails to comply with the court’s order to disclose the evidence at its disposal, it may be justified to create a presumption of non-compliance with the duty of care that such evidence was intended to highlight. The disclosure of evidence is limited to high-risk AI systems for which, according to the AI ACT, specific documentation, disclosure and preservation requirements apply. The causal relationship between the defendant’s fault and the output or lack of output generated by an AI system may be presumed when all of the following conditions are met: the claimant has proved, or the court has presumed, fault on the part of the defendant or a person for whom the defendant is liable, consisting in the breach of a duty of care established by Union or national law, directly aimed at preventing the harm suffered; it is reasonably probable, based on the circumstances of the case, that the negligent conduct affected the output generated by the AI system or its lack of output; the claimant has proved that the harm was caused by the output produced by the AI system or its lack of output.

## 3 Discussion

The convergence of robotics and medicine has opened new frontiers in healthcare, enabling significant advances in diagnosis, surgery, rehabilitation therapies and elderly care (57). Robotics applications in medicine are radically transforming medical practice, offering more precise, efficient and customised solutions (7). In the ever-changing landscape of healthcare, the synergy between robotics and AI is opening up new frontiers, revolutionising medical practice and offering innovative solutions to improve the precision, efficiency and accessibility of care.

Despite the many benefits, there are also challenges to be addressed proactively, ensuring that technology is used responsibly to maximise benefits for patients and society. These include the identification of liability among the various parties involved in medical malpractice cases.

When AI stands between a person’s action or omission and the harm, the particular characteristics of some AI systems, such as the opacity of algorithmic decisions, autonomous behaviour and complexity, can make it extremely difficult for the damaged party to meet its burden of proof. Claimants may face significantly higher initial costs and significantly longer court proceedings than in cases not involving the AI (58). On the other hand, an unfair attribution of blame may occur where physicians are wrongly blamed or blamed for errors or complications that may be beyond their control. In both cases, an injustice would be realised.

In this paragraph, the possible consequences on the determination of medical fault will be analyzed if robots were to be granted electronic personality. Then, the issues related to the potential recognition of strict liability in the context of using AI systems will be discussed.

### 3.1 The status of electronic persons

To address this issue, in 2017 the European Parliament proposed the recognition of a joint human-robot action based on the identification of robots as “electronic persons,” with the possibility for robots to compensate for any damage caused by them (47).

Should the legal personality of robots be recognised, there could be three models in which robots could commit a crime. According to “the perpetration via another liability model” the robot is the means by which the programmer or the end user commits the crime; according to “the natural-probable-consequence liability model,” the offence is caused by the negligent conduct of the programmer or user. In this instance, if the robot/AI committed a different or additional offence, this would constitute an abnormal concurrence of offences; Finally, according to “the direct liability model,” it is viable to identify the robot’s action, its causal connection with the harmful event, and its deliberate intention to perform harmful actions. The conduct may be commissive, such as the movement of the robotic arm, or omissive, such as the inertia of the robotic system. The conscious intention of the robot to commit a crime is constituted through three successive steps: the representation of the real world through the sensor-based acquisition of data and their processing; the ability of machine learning and decision-making systems to anticipate and desire a specific outcome as a consequence of their actions; the occurrence of negligent behaviour because the system does not take into account a probability that it should have taken into account on the basis of the data collected, or in the event of a calculation error during the learning process (59).

According to the European Economic and Social Committee, recognising the “electronic personality” of robots poses a significant hazard (47). However, we believe it is not only a moral, but also a bio-legal hazard, as exposed by the signatories of an open letter to the European Commission on AI and robotics (60).

The “electronic personality” concept is based on the recognition that the robot-autonomous-agent can relate to its surroundings through sensors or the constant exchange of data, learn through experience and interaction, adapt its behaviour and act through physical support. Thus, the recognition of “personality” and ownership of specific rights is conditional on the existence of characteristics such as self-awareness, self-control, ability to relate to others and communication skills. This view is definitely restrictive. Intangible personality. These include the right to one’s name, image, privacy, also understood as control over the circulation of one’s personal data, honour and reputation, personal identity and physical integrity. Recognition of these rights is also followed by preventive protection measures, aimed at preventing the damaging act before it occurs, and restorative measures, aimed at compensating for the prejudice linked to the injury to “personality rights.”

The recognition of an “electronic personality” is also at odds with European legislation according to which the person is at the centre of the initiatives promoted by the European Union and among the fundamental values attributed to him are the right to physical and mental integrity, without distinction (61). In addition, specific rights are attributed to the individual, first and foremost the right to life, from which follow the right to liberty and security, the right to respect for private and family life, freedom of thought, conscience and religion, freedom of expression and other relevant civil rights (62).

Therefore, should the robot-agent be granted “electronic personality,” it would also be necessary to recognise and protect “personality rights” as well as the values recognised and protected by the EU: for example, we should recognise the robot-agent’s right to have its own opinion, express it, decide, without any external, even human, coercion. Otherwise, the right to freedom of thought and expression would be violated. In the healthcare area, therefore, the possibility of a relationship between patient and robot-agent should be recognised, in which the latter is given the right to formulate a medical therapy. The patient’s right to freely choose whether or not to adhere would be the only limit to the medical-robotic act.

Setting the goal of an “anthropocentric” AI, although admirable, is not enough if by this term we only refer to the goal of realising AI-systems at the service of humanity and the common good, with the aim of improving the wellbeing and freedom of human beings. It becomes so when an “anthropocentric” approach expresses the recognition for the human being of a unique and inalienable moral status of primacy in the civil, political, economic and social spheres (63). Indeed, serving others is not an obstacle to the recognition of legal personality, to the same extent as it is not for the human being who is legitimately accorded “personality rights” and who considers service to another human being, to humanity and to the common good as fundamental values. The rights, duties and legal protections enjoyed by a human being due to the recognition of an inherent dignity and value as an individual cannot also be granted to a robot-agent. On these grounds, it is reasonable and well-founded to state that AI systems should be supervised and controlled by a human being, whose task it is to intervene in all potential or actual and concrete cases in which an AI system risks infringing “personality rights” (46, 52). Otherwise, if the robot-agent were recognised as having an “electronic personality,” it would have to be given the same rights and duties as a human being. Consequently, it would be inconsistent to impose prior and continuous surveillance and control over it by human beings.

Among other things, the recognition of “electronic personality” has significant repercussions in terms of legal redress (64). The punishment meted out to a robot-agent as a result of an offence could in no way have any deterrent power towards those tempted to commit a crime or any violation of the law. Similarly, a patient victim of a negligent omissive or commissive conduct by the robot-agent could not receive any form of compensation. Finally, we consider it questionable the establishment of a specific legal status for robots by the recognition of qualities such as autonomy, learning capacity, physical support, relationality. It is not self-awareness, self-control, the ability to relate to others, the ability to communicate that make a human being the holder of inalienable rights. The human being’s status as a person persists even when the ability to think and will is absent, since self-awareness, although fundamental to human freedom, does not constitute the essence of human nature. Even when the intellectual and volitional faculties are irreversibly impaired, the human being remains a person, and human life is not determined by the expression of these faculties (65). For these reasons, we do not believe it is permissible to establish a legal status for robots, allowing sophisticated autonomous robots to be held as electronic persons responsible for the damage they cause and recognising the electronic personality of those that make autonomous decisions or interact independently with third parties.

The centrality of humans in decision-making processes, even with the assistance of AI, is crucial to ensure accountability, ethical considerations, and the integration of human capabilities with AI outcomes. Humans must interpret and wisely apply data provided by AI, as AI lacks the necessary human experience to fully grasp the context and complexities of decisions involving moral and ethical aspects. Additionally, human monitoring can identify and correct potential biases in algorithms. While AI can analyze vast amounts of data and produce rapid results, it can also be influenced by biases or distortions in training data or the decision-making process itself. Human intervention is crucial to understand the specific context in which AI operates, detect discriminatory trends or ethical distortions, and make appropriate changes to ensure AI-driven decisions are balanced, fair, and non-discriminatory. This active monitoring helps mitigate risks of negative impacts from uncontrolled AI use, promoting better adoption and acceptance of technologies that could otherwise engender mistrust or controversy. In summary, collaboration between AI and humans should enhance human capabilities, ensuring AI remains a valuable ally rather than a substitute.

### 3.2 Strict liability

A further critical issue in establishing medical malpractice in the healthcare context is the possible recognition of strict liability for HWs and healthcare organisations. The European Parliament Resolution of 20 October 2020 establishes a strict liability regime applicable to both “front-end operators” and “back-end operators” (54). Given that the “front-end operator” is defined as the natural or legal person who exercises some degree of control over a risk related to the operation and functioning of the AI system and who benefits from its operation, we can in a healthcare scenario consider the “front-end operator” to be both the physician and the healthcare organisation.

According to the AI Liability Directive, the causal relationship between the defendant’s fault and the output or lack of output generated by an AI system can be presumed when specific conditions are met (46), which, translated, in the healthcare context can be summarised as follows: proven for alleged breach of a duty of care on the part of the physician or healthcare organisation; causal relationship between the output or lack of output of the AI system and the negligent conduct of the physician or healthcare organisation. This judgement is made on the basis of probability and not certainty; the patient’s harm is causally related to the output or lack of output of the AI system.

If the harm to a patient was caused by the inadequate, incorrect, or imperfect use of the AI system by the physician or healthcare organisation, or by a misinterpretation of the data provided by the AI system, there is no doubt that liability is attributed to the physician or healthcare organisation. Critical issues arise when the harm to the patient is causally related to the output of the AI system: who is to blame? To the manufacturer of the AI system? To the physician and the healthcare organisation? Or can they both reasonably declare their innocence because the robot-agent has the potential to autonomously increase its capacity through an appropriate deep learning system similar to human neural networks? Can strict liability also be attributed to the doctor and the healthcare organisation in such cases?

In the context of strict liability, the medical doctor or healthcare organisation is liable for harm to the patient regardless of fault, but under the rule of risk. Neither of them can be exempted from this liability, except exceptionally by proving that the harm occurred as a result of a fortuitous event. Strict liability is a type of liability designed to protect the injured party by requiring him to prove only the damage and the causation.

One limitation to the use of strict liability in the healthcare sector is the recognition of the existence of a joint human-robot activity based on two essential interdependent relationships, namely predictability and directionality (46). If we were to admit the existence of a joint human-robot action, why should strict liability of the medical doctor or healthcare organisation be recognised?

Strict liability obliges the victim to prove causation and the defendant to prove that the harm occurred due to a fortuitous event. It is required to resort to a logical procedure called the “but-for test”: for comissive conduct, a “but-for test” will be carried out based on the mental elimination of conduct from the causal course. If, by eliminating the conduct, the event would have occurred anyway, then that conduct cannot be said to have caused the event. With regard to omissive conduct, the conduct that should have been performed will be added to the causal course and it will be ascertained whether or not through this conduct the event would have occurred (66–68).

The logical reasoning just outlined is the foundation of the study of causation in both civil and criminal liability, but it is very unlikely to find application in cases where AI stands between a person’s action or omission and the harm. In fact, the latest machine learning models are like “black boxes,” as their extremely complex structure prevents users from understanding the process by which an AI system processes data to arrive at decisions (69, 70).

It would also preclude any possibility of analysing the gradation of fault by means of a procedure for assessing the seriousness of the physician’s misconduct or negligence and the corresponding level of legal liability.

Although aimed at harmonising legislation, both the European Product Liability Directive and the Directive on Liability for AI fail to close potential liability gaps (71).

We are experiencing a hybrid phase in which AI and medicine are increasingly joining forces to improve the diagnosis, treatment and management of diseases. However, at present we have many critical social, ethical and legal issues to study, address and overcome (72–74). The accelerated development of AI is far exceeding the capacity of the legal framework to fully understand its implications (75).

So, what can be done? First of all, the centrality of the relationship between physician and patient in the healthcare context must be reaffirmed. On the one hand, it is the physician’s sole task to guide the diagnostic and therapeutic process, using their expertise and experience. On the other hand, the patient has the fundamental right to consciously participate in the proposed treatment, fully understanding its implications, and actively contributing to decisions concerning their own health. This balance between medical expertise and patient autonomy is essential to ensure effective care, while respecting the dignity and self-determination of each individual. With this in mind, it is crucial to avoid any distortion of automation that could undermine human autonomy by interfering with the decision-making process (4). This risk becomes even more significant considering the existence of known algorithmic biases in AI-supported clinical decision-making (76).

Therefore, in line with the current state of technology, an AI system should not be considered differently from radiology devices. Radiology as a diagnostic support tool offers multiple benefits, including more accurate and timely diagnosis, effective monitoring of treatment response and guidance during invasive procedures. However, it is essential to correctly interpret the diagnostic images and integrate them with other clinical information in order to ensure a complete and accurate assessment of the patient.

Therefore, in determining liability for medical malpractice, even when using AI systems, it is still necessary to consider the scientific dimension of causation, integrating the “but-for test” with the “covering-law model.” This approach makes it possible to assess the physician’s actions on the basis of universal laws and statistical-quantitative and epidemiological rules (66–68).

Despite the revelation of the limits of linear causality by contemporary physics and mathematics and by cognitive relativism (77–79), the but-for test causality model integrated with the covering-law model can still effectively address the challenges posed by AI-integrated medicine and assist in the search for judicial truth.

In summary, the introduction of a regime of strict liability would imply that doctors and healthcare organisations are held accountable for the correct and safe use of AI systems in the healthcare sector. However, we believe that even when AI systems are used, they should be contextualized within a joint human-robotic action. Such action should be evaluated through the forensic science methodology.

## 4 Conclusion

Technological progress has played a fundamental role in medicine, revolutionising virtually every aspect, from diagnosis to therapy, and even data management. The introduction of robotics and AI have the potential to redefine the landscape of medicine, radically transforming clinical practice and improving the lives of millions of people worldwide. However, while in the past, technology’s role has been explicitly to assist in medical procedures, today, for the first time in history, new technologies can potentially enter the physician’s decision-making process to the extent of replacing it.

We do not believe that an approach to analysing the problem based on a division between those who take a catastrophic attitude and fear professional deskilling and those who are carried away by easy enthusiasm is useful.

The goal of medicine is the patient’s health, so if in the future robotics and AI were to pursue the objective of patient health better than the activity performed by a human being, and this is supported by solid scientific evidence, so be it.

However, we are currently in a hybrid phase where the last mile, i.e., the decision, lies with the physician. And it is at this stage that we have to deal with, and it is at this stage that those who deal with medical liability should, on the one hand, guarantee fair compensation to patients who are victims of harm, and on the other hand that doctors and health organisations should not be found objectively guilty because they have made use of AI devices.

As it turns out, the current European legislation aimed at harmonising legislation in this area leaves some questions unanswered. However, precisely on the basis of the European regulations, we believe that a specific regulation concerning medical liability in cases of the use of robotics and AI in medicine should be drawn up. To this end, it will be necessary to analyse the risk of the use of AI in health care and then assess the specific features with which AI is used. The integration of robotics in healthcare offers significant advantages, but it also presents significant ethical and medico-legal challenges. Addressing these challenges requires deep reflection and collaboration among healthcare professionals, legal experts, legislators, and stakeholders. Only through a holistic and ethically based approach can we maximise the benefits of robotics in healthcare, while ensuring the safety, privacy, and well-being of patients.

## Data Availability

The original contributions presented in the study are included in the article/supplementary material, further inquiries can be directed to the corresponding author.
